# The Cod and the Cut: Intra-Active Intuitions

**DOI:** 10.3389/fsoc.2021.724751

**Published:** 2021-10-13

**Authors:** Tilman Hertz, Maria Mancilla Garcia

**Affiliations:** ^1^ Stockholm Resilience Centre, Stockholm University, Stockholm, Sweden; ^2^ Socio-Environmental Dynamics Research Group (SONYA), Université Libre de Bruxelles (ULB), Bruxelles, Belgium

**Keywords:** intra-action, causality, cut, Baltic cod, material-discursive arrangement, performativity, process-relational perspectives, intuition

## Abstract

Interest in causality is growing in sustainability science and it has been argued that a multiplicity of approaches is needed to account for the complexities of social-ecological dynamics. However, many of these approaches operate within perspectives that establish a separation between what has causal agency and all the rest, which is relegated to the role of background conditions. We argue that the distinction between causal elements and background conditions is by no means a necessary one, and that the causal agency of background conditions is worthy of investigation. We argue that such conditions correspond to what Karen Barad has called a “cut”: a specific determination of the world (or part of it) respective to another part, for which it becomes intelligible. In this sense, most approaches to causality so far operate from “within” particular cuts. To illustrate this, we focus on the paradigmatic case of the Baltic cod collapse in the eighties. This case has been extensively studied, and overfishing has been identified as a key cause explaining the collapse. We dig deeper into the conditions which characterized fishing practices in the run-up to the collapse and uncover the separation between the social and the ecological that they enforce by encouraging policies to increase productivity under the rationale of national “development”. We then re-examine the case from a process-relational perspective, rejecting the separation of nature from society. A process-relational perspective allows us to consider relations as constitutive of processes through which what exists becomes determinate. For this purpose we use the concepts of intra-action (co-constitution of processes) and of performativity (determination of language and matter within processes). We complete our conceptual framework by drawing inspiration from pragmatist philosophers and suggest that the concept of intuition can constitute an alternative to untangle causal dynamics and explain social-ecological phenomena beyond the cause/condition dichotomy. This article seeks to fulfil two objectives: firstly, to question the thick boundaries between conditions and causal elements that explain the processes in which social-ecological systems evolve; secondly, to provide a different approach to transforming a social-ecological system.

## Introduction

Truth, according to Foucault, depends on “the instruments required to discover it, the categories necessary to think it, and an adequate language for formulating it in propositions” (Foucault et al., 2008). The question of Truth is thus not a question of a supposedly right access to reality but a question of compliance with a technology of demonstration (Lorenzini, 2016). A technology of demonstration is a material-discursive arrangement of processes and practices which *produces* truth. If, as (Heidegger, 1927) suggests, the question of Truth is intimately connected to the question of Being–Truth as an unconcealement of Being–then Being itself is a product of a material-discursive arrangement. To make sense of the process of unconcealement we draw on Barad’s (2007, 2003) work: a material-discursive arrangement realizes a “cut” where a “part” of the world becomes intelligible to another “part” of the world. She calls these arrangements “apparatuses”. Apparatuses *perform* reality.

We argue that paying attention to the apparatus that produces a particular “cut” provides a fresh angle for understanding sustainability problems. In this paper we take the example of the collapse of the Baltic cod in the 1980s. To date, there exists a manifold of studies on this social-ecological phenomenon which have found many different causal explanations, from different disciplinary angles. We argue that these perspectives all operate from within a particular “cut” within which entities and their properties have already become determinate. We argue that a cut is not something necessary, it is not something inherent to *nature* but something actively enforced or performed. This allows us to revisit the cod collapse focusing on what other approaches relegate to “conditions” and examine how they might be causally relevant.

The emergence of a particular cut can be conceived of as a “gesture” (Debaise, 2017) and we single out two major processes that oriented (and continue to orient) this gesture, 1) the bifurcation of nature dating back to modern philosophers and, 2) the continuous development and application of disciplinary approaches inhering from the bifurcation gesture the separation of nature from society, particularly in natural resource economics, international relations and fisheries science. Our emphasis on performativity–inspired by our reading of scholars as diverse as Butler (1988), Derrida (1988), Barad (2003), Callon (2006), Butler (2010), MacKenzie and Bamford (2018), MacKenzie et al. (2020a, 2020b)–leads us to an understanding of causality that is best expressed in terms of intra-actions (Barad, 2007), as opposed to the more familiar understanding of causality in terms of regularities between events. We end this paper by highlighting the important role that “intuition” plays for an intra-active analysis and show how our analysis points to very different ways and means to transform a system–as opposed to those ways and means that would arise from an analysis carried out from within a cut. Next to presenting, applying and discussing an understanding of causality in terms of intra-actions, this paper thus seeks to contribute to the wide and diverse literature on sustainability transformations, especially to those strands that focus, for example, on the transformative powers of novel narratives and relationships (see e.g., Leichenko and O’Brien, 2019; O’Brien et al., 2019).

## Cuts

### Apparatuses Produce Cuts Within Phenomena

The concepts of apparatus, cut and phenomenon come to us from Karen Barad who is part of the new materialist movement which–albeit very heterogeneous–shares a commitment to critical inquiry beyond constructionist frameworks. “Language has been granted too much power” is the sentence she begins one of her works with (Barad, 2003) and points to the need to put matter equally in focus. As a transdisciplinary field of inquiry new materialism draws, among others, from the fields of feminism, philosophy, science studies, and cultural theory (Yi Sencindiver, 2017). Barad’s own approach stems predominantly from the field of philosophy and quantum physics, paying particular attention to the writings of quantum physicist Niels Bohr. The ideas presented below are not specific to these fields, nor would it be adequate to say that they emerged solely from Barad. In sociology for example, scholars as diverse as Norbert Elias and Niklas Luhmann have developed ideas during the last century that resonate with the body of ideas presented here. For example, Luhmann’s concept of difference, or distinction (Luhmann, 2006), which recently attracted increased attention also in the English speaking world, is of great interest when applied to social systems (Seidel and Becker, 2006). The reason why we chose to focus on Barad’s cut to convey our ideas, however, is that the “cut” is a concept that applies distinctively beyond human-life worlds. Accordingly, we argue that it might be more adequate for the inquiry of social-ecological systems. Also, as we will argue below, it does not presuppose the existence of human actors as separate identities, that is, as psychic embodied systems (Willert, 2019), thus allowing for more varied ways in which the “social” and the “ecological” might materialize.

Barad (2003, 2007, 2012) introduces us to a view of reality where there is a fundamental entanglement between agencies of observation, matter, and discourse. Entanglement implies that these do not pre-exist but emerge within what Barad calls a “cut”. Within a cut “ “part” of the world becomes determinately bounded, propertied (and meaningful) in its emergent intelligibility to another “part” of the world” (Barad, 2007). Her work is inspired by Niels Bohr’s work in the field of Quantum Physics. It focused on whether an element such as an electron is a particle or a wave is determined by how the experiment is done. The way the experiment is designed determines the properties of the electron (properties of particles, or properties of waves). Crucially, these two understandings are mutually exclusive: it cannot be both a particle and a wave at the same time. However, this is only problematic if one takes the electron, as an independent object, to be the objective referent. Instead, “the actual objective referent is the phenomenon (...) And so the fact that its [the electron’s] ontology changes when we change the apparatus is not a surprise, because we are investigating an entirely different phenomenon.” (Barad, 2012).

Particular attention should be drawn to what Barad (2007) calls “apparatuses” which are those material-discursive arrangements that produce phenomena. They are material-discursive because these two dimensions are entangled in the sense that material things, such as instruments, and discursive ones, such as concepts and ideas, are only meaningful in relation to each other. Apparatuses are not mere devices whose aim is to discover a pre-existing reality, but they are productive of reality. Put differently, they are *performative* (Barad, 2003). Apparatuses “provide the conditions for the possibility of determinate boundaries and properties of “objects” within phenomena” (Barad, 2007) where “phenomena” refer to the ontological inseparability of objects, apparatuses as well as agencies of observation. Put differently, apparatuses produce phenomena but are at the same time ontologically inseparable from, or entangled with what they produce. Another way of making this point is to say that apparatuses effectuate, or realize cuts within phenomena. Cuts refer to distinctions, that is, when objects, their properties as well as agencies of observation become determinate, or materialize. Indeed, it is important to emphasize that an agency of observation is not separate from the phenomenon within which a cut is disclosed, but that it is also a product of the apparatus. Using Byrant’s (2008) words, “a point of view or a perspective [a cut] is not something that belongs to a subject [agency of observation], but rather a subject [agency of observation] belongs to, occupies, or is occupied by a point of view or perspective [a cut]”. In this sense one can say that the cut orders the world in a certain way, in a way that facilitates some connections and hinders others.

The material-discursive arrangements producing cuts make distinctions that inevitably direct phenomena to certain paths and not others. Concepts such as “humans” and “nature”, or “social” and “ecological” introduce fundamental distinctions on which other distinctions might be built, such as “resources” or “optimization”. This in turn might make it difficult for other phenomena, unrelated or opposite to optimization, for example, to materialize. For instance, under a perspective of optimization it might be impossible to perform cultural or spiritual meanings (Mancilla García et al., 2020). Our reading of Barad’s statement “Discourse is not what is said; it is that which constrains and enables what can be said. Discursive practices define what counts as meaningful statements” precisely captures this idea (Barad, 2003). Yet, as mentioned above, discourse is entangled with material counterparts. For instance, particular instruments, procedures, or devices, e.g., those involved in regulating or monitoring the use of a particular resource are intimately tied to concepts such as optimization, in that, through their materiality, they make sense of what optimization can concretely refer to or mean. “Matter is not a thing, but a doing (...) Material constraints and exclusions and the material dimensions of regulatory practices are important factors in the process of materialization”, Barad notes (2003). Material-discursive practices together produce phenomena within which cuts realize and within which further, specific cuts–or specific distinctions–might materialize. We refer to this as the *unfolding* of a cut.

The picture that emerges from our discussion is thus one where the social and the ecological do not pre-exist *qua* social and ecological but where this distinction is produced, and where the social and the ecological can materialize in very different ways, ranging from the one or the other being conceived of as a driver, or as coupled, or as the social being embedded in the ecological (see Schlüter et al., 2020). Such distinctions can themselves take the role of material-discursive background practices that condition the development of further cuts, such as can be found, for example, in practices of different disciplinary approaches that build on the same fundamental distinction of the social and the ecological. In what follows we provide two different examples of phenomena where the social and ecological become determinate in different ways by two distinct material-discursive apparatuses producing them.

Consider a situation where members of a community come together to discuss, develop and implement practices that would allow them to sustainably diversify their livelihoods. They turn to an international NGO for advice. The NGO recommends exploring and exploiting adjacent fishing grounds. It supports this process and provides a best-practice guide sketching out a step-by-step procedure to develop sustainable practices for fishery management in a participatory and inclusive manner. The guide introduces the community to the state-of-the art, Western University type of scientific and managerial knowledge about the issue and conceptualizes it as a collective action problem framed in terms of concepts such as agents, resources, optimization, reproduction, fishing seasons etc. In line with the procedure sketched out in the best-practice guide, the community agrees on the type of gear for each fish species, on the type of vessels to use, on fishing seasons and amounts of fish to be captured per fisherman. In implementing these practices–which become part of a wider material (e.g., gear, vessel) as well as discursive (e.g., conceptualizing fish as “stocks” and “resources”, optimization, fishing seasons) apparatus–the community members and the fish populations that participate in this particular phenomenon become determinate as entities with properties of different types. Fundamentally, for example, fishers become individual subjects, whereas fish become objects, that is, resources of pure instrumental value for the subject. Yet, there is no necessity in this organization of socio-environmental dynamics. In other words, other material-discursive organizations arrangements or organizations of processes and relations might lead to different dynamics (see also Schlüter et al., 2020).

**FIGURE 1:**
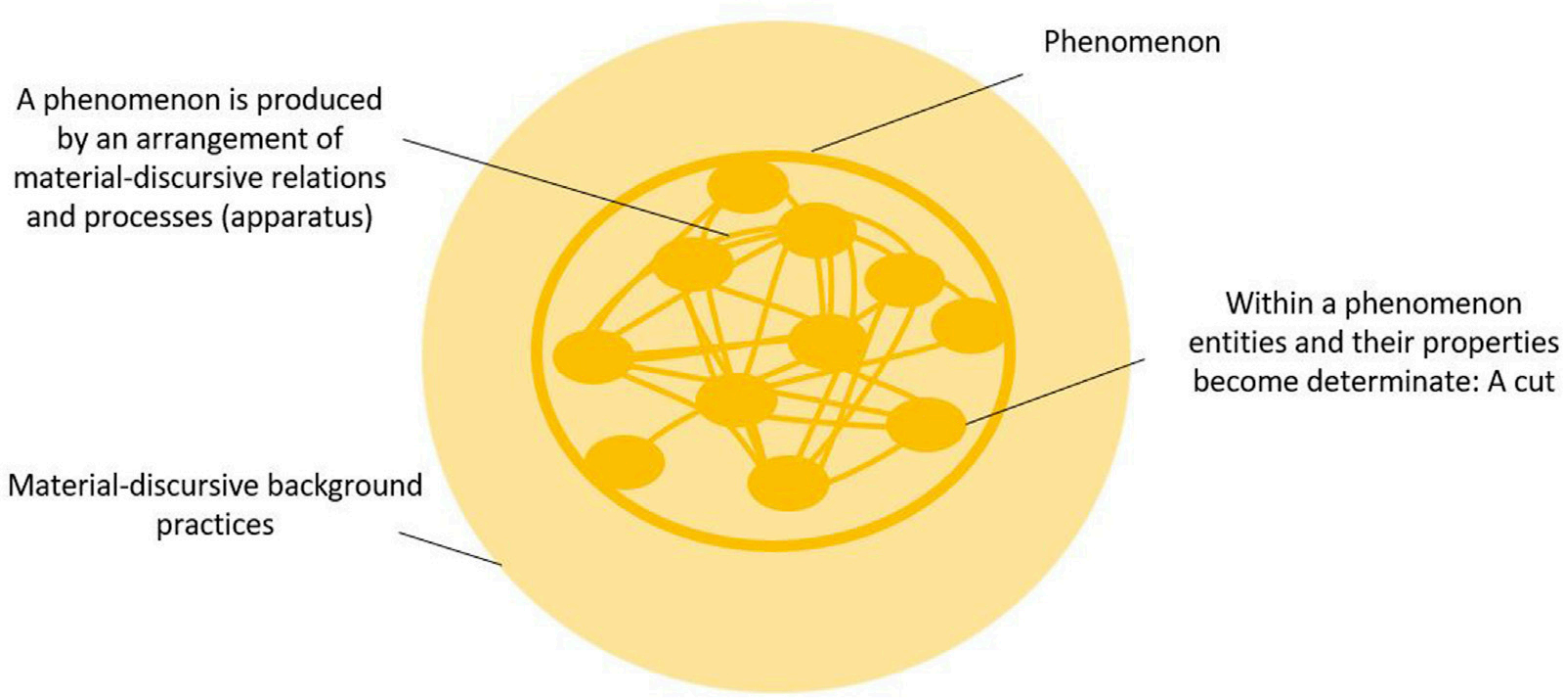
Cuts, phenomena and apparatuses - adapted from Schlüter et al. (2020).

Consider the “ayllu”, a quechuan word that Andean anthropology has often translated to refer to a form of community organization. Yet, anthropologist Marisol De la Cadena (2015) describes the ayllu as a phenomenon within which entities and their properties become determinate in ways that are different to a simple type of collective organization. She argues that “ayllu” is something that “takes place”, and by taking place, people and the land exist together as what she calls earth-beings. Ayllu “taking place” is the phenomena within which entities become determinate, but in radically unfamiliar (to Western-formed minds) ways: humans are not subjects and the mountain is not an object, but they become determinate in ayllu as earth-beings. Here we have a very different arrangement of relations (“ayllu”) that, once actualized, realizes a phenomenon within which entities become determinate in a very different way than in the first example (Schlüter et al., 2020) (Figure 1).

Two additional points to finalize this short discussion of apparatuses and their performative power. First, what is performed is not random–the possibility space for material-discursive apparatuses to emerge and unfold is determined at any moment by what Simondon (1995) calls pre-individual singularities, and which refer to realities *before* entities and their properties become determinate within particular phenomena (see also Debaise, 2012). Second, not only does a cut unfold, which as we have seen above is a process that we can understand as subsequent cuts building on each other, but performative processes are also always “impure” (Derrida, 1988). They escape “from the will and intention of that which created it” to borrow a sentence from Morin (2007) and demonstrated by MacKenzie and Bamford (2018). There is always a sense in which “the world kicks back” (Barad, 2007). Indeed, in their actualization, performative processes are in constant re-negotiation. Thus, strictly speaking, there is no “one” cut, but as many cuts as there are agencies of observation. Yet, from a pragmatic perspective we can say that similarities exist across cuts (which is a point we will return to below when we discuss how a cut is disclosed in a shared narrative), and this allows us to refer to the emergence of the cut.

### Cuts and Causality

How can we describe and qualify the ontological view of reality described above? For sure it is not the familiar one, where elements exist *as* elements independently of us in a world beyond our understanding. Instead elements materialize within phenomena. To capture this idea Barad (2007, 2012) introduced the notion of intra-action in view of opposing it to the notion of interaction. In her own words “ “intra-action” signifies the mutual constitution of entangled agencies. That is, in contrast to the usual “interaction,” which assumes that there are separate individual agencies that precede their interaction, the notion of intra-action recognizes that distinct agencies do not precede but rather emerge through their intra-action. It is important to note that the “distinct” agencies are only distinct in a relational, not an absolute, sense, that is, agencies are only distinct in relation to their mutual entanglement they don’t exist as individual elements (Barad, 2007).” It is not so much that individual agencies do not *not* exist, but that they are not individually determinate. Individual agencies, she goes on, “only exist within phenomena (particular materialized/materializing relations) in their ongoing iteratively intra-active reconfiguring” (Barad, 2012).

This challenges the familiar understanding of causality. Indeed, causality is typically stated in terms of regularities between events. This dates back to David Hume who argued that causality is nothing but a constant conjunction between events. Events, in turn, are conceptualized in terms of causal agents of whatever type (element, entity, actor, process) “doing” things. We speak of a cause and effect relationship whenever events regularly succeed each other–within certain background conditions. The distinctive characteristic of this account is that causal agents exist *qua* causal agents before interacting with each other. How to understand causality in a world where causal agents do not pre-exist as causal agents, that is, in an intra-active world? The notion of intra-action provides a redefinition of causality, not as a regularity *between* events but as a repeatability of a material-discursive arrangement of relations *producing* events (which we interpret here as phenomena). The appropriate referent for approaching causality are, as we have discussed in the previous section, phenomena and not particular pre-existing causal agents such as entities and their properties. With this in mind we turn to Rouse (2002) who notes: “That a particular intra-action is causal indicates that under the right circumstances its pattern would recur, but there need be no actual regularity that it instantiates”. This is because the repeatability of a phenomenon is essentially *normative* instead of *regular* (Rouse, 2002). Put differently, those patterns that we have called material-discursive arrangements “articulate the world in ways that are semantically and epistemically normative” (Rouse, 2009), and not simply regular (Rouse, 2002). Thus, no *actual* regularity needs to be involved in the production of a phenomenon.

Implicit in this approach is that a very same phenomenon can be repeated by realizing the very same semantic and epistemic (i.e., material-discursive) arrangement of relations. An analysis into what produces the cut within which the cod collapse occurred is therefore also a causal analysis. One immediate implication is to revisit the familiar distinction that is often made in a causal analysis between *causes* and *background conditions*. Not surprisingly there are many discussions around this distinction but for the purpose of this analysis we take conditions to be those elements that necessarily need to be present for a cause and effect relationship to realize. How concretely this distinction is made in an analysis depends on a variety of factors, such as the research question, pragmatic reasons (e.g., availability of data), or on a particular disciplinary angle. Yet, fundamental commitments, such as commitments to particular entities and properties as they are disclosed in a cut, are rarely questioned. They are most of the time relegated to background conditions.

The argument put forward in this paper is precisely that a cut is not something inherent to reality but something actively enforced. The philosopher John Stuart Mill already observed that the distinction between causes and background conditions was spurious: “The real cause is the whole of these antecedents; and we have, philosophically speaking, no right to give the name of cause to one of them exclusively of the others” (as discussed in Rockwell, 2016). We hypothesize therefore that we should bring these background conditions to the front: Not only will this provide a fuller picture of the causal processes at work but it also might provide other avenues, perhaps novel ones, to intervene and transform a system.

In what follows we present some of the existing causal analyses of the cod collapse and show that many of these operate from *within* an existing cut. We will proceed by defining the performative, material-discursive arrangements that realize this cut: 1) The bifurcation of nature (Whitehead, 1919) which realizes an ontological distinction between humans and nature and, building on this distinction, 2) disciplinary frameworks from international relations, economic science and fisheries science that guide the development of management approaches for the Baltic cod. We will end by discussing the causal role of this arrangement for the collapse of the cod stock.

## The Cod Collapse in the Baltic Sea

### What the Existing Analyses Have in Common

In the 1980s the Baltic cod fishery was characterized by high cod biomass and catches, the so-called cod boom (henceforth the “cod boom”). Cod stocks collapsed in the mid 1980s and gave way to a sprat-dominated ecosystem with low cod abundance (Möllman et al., 2008). The collapse has been extensively researched and many–but not mutually exclusive–reasons for it have been provided. In the 2014 report of the Baltic Fisheries Assessment Working Group, the International Council for the Exploration of the Sea (ICES, 2014), for example, identifies several factors, among which an increase in fishing (via traditional bottom trawl fishing as well as novel gillnet fishing) and a decreasing reproductive success of the cod due to a limited inflow of oxygenated, saline water from the North Sea. Bagge et al. (1994) acknowledge adverse effects of natural factors such as eutrophication for recruitment (number of fish entering a fishery) and suggest a link between (over) fishing and (shrinking) spawning stock sizes. The recruitment failure was also highlighted by Köster et al. (2005) and Möllmann et al. (2008) who suggest that climate-induced hydrographic change led to high egg and larval mortality (see also Lindegren et al., 2009). Lade et al. (2015) put forward integrated social-ecological explanations with a focus on their intertwined dynamics, such as psychological, economic and regulatory aspects and how these influence the interactions between e.g., cod and sprat. It is not possible here to give a complete account of the existing literature of the 80s cod collapse in the Baltic sea. All of these have contributed to providing an ever richer, causal picture of the collapse of the Baltic cod in the 80s and we would like to contribute to this endeavour by drawing attention to an aspect of the problem that might be less often in the focus.

While the above analyses all focus on different aspects of the collapse, we argue that they are carried out in large parts from *within* the same cut. System boundaries might be set differently (in terms of geographical and time scales), and the focus realm of the studies might differ (e.g., social or ecological or social-ecological); there might also be different units of analysis (e.g., individual agents, or entire populations) and different explanatory elements put forward. However, all these studies acquire their *intelligibility* and *significance* in the context of the cut. Within a cut ““part” of the world becomes determinately bounded, propertied (and meaningful) in it’s emergent intelligibility to another “part” of the world”, Barad (2007) notes. But where “is” the cut, how can we make sense of it? We argue that a cut is disclosed in what Rouse (1990) calls a shared narrative. This narrative is rarely foregrounded, but researchers drafting such studies can pre-suppose that their peers know and understand what they are talking about, irrespective of above named differences. Importantly, while such a narrative might be shared, this does not mean that there are no differences between analyses. Clearly there are differences in how, for example, an economic scientist and a marine ecologist go about when analyzing a fisheries issue. The elements they study, as well as the properties and capacities that are of interest might not be the same. The ecological fish is a different fish than the fish of natural resource economics with a whole different body of material discursive practices producing it. This echoes a point made above: strictly speaking, there is no *one* cut, but as many cuts as there are agencies of observation. Accordingly, there is also not one single narrative but rather a field of narratives which “displays a constant tension between a need for a coherent, shared understanding of the field and the incoherence threatened by divergent projects and interpretations” (Rouse 1990). However, when researchers from different scientific traditions work hand in hand in view of a common goal, such as the management of a fish stock, we argue that they manage to realize this shared understanding that characterizes a cut.

We now identify the main elements that produce the cut we are interested in. We can qualify this cut as the gradual “becoming intelligible” of the modern cod fishery management in the Baltic sea. We argue that this cut is produced by apparatuses that build on each other. These are of two types: A metaphysical one, the so-called “bifurcation of nature” (Whitehead, 1919) heralding “modernity” and, based on it, models and approaches of more disciplinary nature that joined hands realizing the modern cod fishery in the Baltic sea, such as those natural resource economics, international relations and fisheries science.

#### The Bifurcation of Nature

As Debaise (2020) observes: “Modernity is an invention. It is an invention that coordinates all experience and gives a vision that the moderns have of the world and the place that they want to take in the world”. Bruno Latour traces this vision back to what Whitehead (1919) has called the bifurcation of nature. It is “what happens whenever we think the world is divided into two sets of things: one which is composed of the fundamental constituents of the universe—invisible to the eyes, known to science, yet real and valueless—and the other which is constituted of what the mind has to add to the basic building blocks of the world in order to make sense of them (Latour 2014). There is thus an ontological distinction between what is “real” (fundamental constituents of the Universe: matter) and what is “unreal” (e.g., what the mind has to add to it, which “holds within it the greenness of the trees, the song of the birds, the warmth of the Sun, the hardness of the chairs, and the feel of the velvet” (Whitehead, 1919). A manifold of other dichotomies are derived on the basis of the mind and matter bifurcation, for example, subject and object, objective and subjective, and nature and culture.

In a bifurcated reality nature is reduced to its physicality. Nature is relegated to the realm of the matter, to the realm of objects, to a senseless, valueless, and purposeless resource. Purpose is to be found within the mind. This bifurcation of nature does not represent reality. Instead, in its performative function, it creates reality. The philosopher Martin Heidegger (1954) talks about a particular mode of orientation towards Being in which “nature reports itself in some way or other that is identifiable through calculation and that [it] remains orderable as a system of information”. He identifies the human strive for controllability and predictability as being major drivers for the reduction of nature to its physicality, because such a reduction allows one to express nature in terms of its primary qualities such as motion, extension, or solidity. What is more, an understanding of nature in those terms is a pre-condition for much of scientific practice as we know it (and not the result of it). Heidegger (1954) shows how classical physics is dependent on this mode of orientation to work and not the other way round. Classical physics can only do what it does because of this mode of orientation towards nature which is therefore a pre-condition for some of our scientific practices to develop.

Whitehead (1925) observed that this organization of society and science worked, and it did so in an astonishing way: “We must note its [the bifurcation of nature] astounding efficiency as a system of concepts for the organisation of scientific research. In this respect, it is fully worthy of the genius of the century which produced it. It has held its own as the guiding principle of scientific studies ever since. It is still reigning. Every university in the world organises itself in accordance with it. No alternative system of organising the pursuit of scientific truth has been suggested. It is not only reigning, but it is without a rival”.

We argue that the enormous scientific and technological advancements that were achieved on the basis of a reduction of nature to its physicality contributed to *creating reality in its image*. The power to control and to predict, and the manifold achievements it allowed for, are significant characteristics of what we call “modernity”. We can understand the drive, the desire for controlling and predicting leading to these achievements as having territorializing effects in the sense discussed by Deleuze and Guattari (1987). In other words, we can understand them in terms of processes that keep this model of reality in place and stabilize it. The bifurcation of nature thus produces a particular understanding of nature and the real danger is, according to Heidegger (1954), that it will become so pervasive as to conceal other ways of understanding nature.

#### The Role of Disciplinary Models and Approaches From Natural Resource Economics, International Relations and Fisheries Science as Applied to Fisheries Management

The bifurcation of nature, as a metaphysical model of reality, orients the development of scientific practice, as Seckinelgin (2009) notes: “Through the conceptualization of the subject-object relationship, realities in relation to human beings are being produced (...) This framing structure, which gives meaning to the location of the subject, humankind, is taken to be a cosmological framing. It is believed that the development of discursive disciplines and actions they prescribe take place on the basis of a given cosmology”. It is in this sense that the distinction we have made in the section above (*Apparatuses Produce Cuts Within Phenomena*) between, on the one hand, material-discursive background practices realizing a fundamental cut, and, on the other hand, further specific material-discursive practices realizing further cuts on its very basis, should be understood. Disciplines that joined hands in realizing the modern cod fishery in the Baltic sea, such as natural resource economics, international relations and fisheries science, *require* this cosmology (the bifurcation of nature) for their functioning, but they also contribute to a further unfolding of the cut, each in their own way.

The performative power of economic models, for instance, derives from the fact that they “work” which means that they generate profit for powerful actors or groups. When Holm (2020) discusses the work of Michel Callon he notes “While economic man, say, at the outset is a purely abstract notion of economics it gradually becomes true because powerful actors manage to reconstitute the world in its image” (...) “Callon rejects the idea that economics–the theoretical abstractions of economists–can be separated out radically from the practical workings of the economy (...) Economists do not study the economy, they *perform* it”. This ongoing performativity, Callon (2007) notes, is achieved by processes of economization which refer to those processes that render actions, things and processes economic. In the area of natural resource economics such processes refer in the most general sense to the “becoming-resource” of something, by setting up markets for it by defining property rights, rules for access, pricing and trading, as well as bodies responsible for governing and overseeing transactions.

A particularly striking example of a process of economization in fisheries management is the establishment of Exclusive Economic Zones (EEZ) decided at the UN conference on the sea in 1972. EEZ are defined in geographical terms (up to 200 nautical miles from the coast) and detail a state’s sovereign rights over this area, that is, by giving it the “sovereign rights for the purpose of exploring and exploiting, conserving and managing the natural resources, whether living or non-living, of the waters superjacent to the seabed and of the seabed and its subsoil” (UNCLOS). The linguistic reformulations in the UNCLOS text “of marine resources as “agent” and the new relationship established are the means through which an EEZ area becomes functional as the industrial zone” as Seckinelgin (2009) notes, implying a redefinition of the ecological question in economic terms. This type of policy imposes a land-inspired zonification of the ocean and distributes roles and identities by performing certain boundaries (Boucquey et al., 2016).

But how to make sense of an *optimal* resource use, such as fish? It is here that fisheries science provides us with concepts such as Virtual Population Analysis (VPA) and Total Allowable Catch (TAC). A VPA is a stock assessment technique which estimates the fish population over a given time period. In turn TAC defines, on the basis of the VPA, how much could be taken from a particular stock over a given period without it collapsing. These two concepts go hand in hand as Holm and Nielsen (2007) point out. For these assessments to work, fish need to be expressed in terms of their physicality, i.e., in terms of aspects that could be captured in physical terms– and this is precisely what the bifurcation of nature laid the groundwork for. A VPA, for example, expresses fish in terms of concepts such as growth rates, natural mortality and fishing mortality. This gives a sense of “control” over the resource as the basis for realizing the economic rationale. As Johnsen et al. (2009) observe, “through the TAC-machine, the wild unmanageable fleshy fish of nature can be translated into a domesticated manageable object”.

When natural resources are an international management issue we touch upon the domain of international relations and the purpose of optimizing the economic rationale in an international setting. Seckinelgin (2009) notes “The ecological question can be only answered by the discipline of International Relations after the disciplinary framework redefines what is being asked; in other words, it can only respond if it understands the problem as a failure in the steering mechanisms of international cooperation, regimes or bargaining strategies”. Examples of such international mechanisms are fishing quotas for involved countries, be they for individual fishing vessels (e.g., Individual Vessel Quota–IVQ) or transferable (e.g., Individual Transferable Quota–ITQ). Via such mechanisms of international cooperation, fish are expressed in quotas that can be owned and traded before even being fished.

The argument is that models and approaches from natural resource economics, international relations, and fisheries sciences are not representations of a pre-existing reality that they are meant to mirror. Rather, there is talk about “reconstitution” of the world in the image of the economist, or “redefinition” of an ecological problem by the disciplinary framework of international relations. These models and approaches are the apparatuses that produce the phenomena of international fishery management within which entities and their properties become determinate. Within this cut the “fish-asfit-for management” [term coined by Holm and Nielsen (2007)] comes to life as the result of “the construction and stabilization of a heterogeneous network, tying the fish in with fishermen, echo integrators, log books, legislation, computers, bureaucracies, mathematical formulas, and surveillance procedures. It is within such a network that the fish-asfit-for-management springs to life, as a true cyborg: part nature, part text, part computer, part symbol, part human, part political machine. It [...][is] a story about entities with variable ontologies, about actors that become what they are as their relationships with other actors stabilize. It [...][is] a story about performation, about theories of fish and fishermen that make the leap from flat paper surfaces into reality” Holm and Nielsen (2007).

In its performative unfolding, the intra-active nature of the cut is pervasive in its material-discursive dimension. Consider, for example, the materiality of extractive devices such as bottom trawling nets used in fishing which are only meaningful within a discursive scheme where nature is purpose–and valueless, and where nature is seen as a pure resource. Or consider, for instance, the concepts of growth rate, natural mortality and fishing mortality. These concepts become meaningful only within a network of material devices and technical procedures such as echo lots, log books and modeling procedures that are geared to it. In a certain sense, they “express” these concepts. At the same time these material devices and technical procedures become meaningful only as a counterpart to the discursive dimension. In this way the cut orders the world in a certain way, in a way that facilitates some connections and hinders others.

#### Increasing Productivity for Economic Development and the Collapse of the Cod

This is the story of modern fishery management. How did it contribute to the collapse of the Baltic cod? Here, we argue that a territorializing process of economic nature that we can characterize as emanating from a drive for increasing productivity in the name of national development led to the collapse of the Baltic cod. This process–on its own–does not *explain* the collapse of the cod. Instead, the argument of this paper is that this process of increasing productivity as way and means to realize economic development of nation-states is *only intelligible within this particular cut*, that is, only where the bifurcation of nature has already set the scene for a subject/object distinction, and where nature is relegated to the realm of the object as intrinsically purpose–and valueless. The following paragraphs describe how this drive for ever increasing productivity led to practices that further territorialized the cut which in turn led to the collapse of the Baltic cod.

While up until the second half of last century there was no regional body authorized to formulate and enforce cod fishery regulation, there was a body that advised governments on the matter, the International Baltic Sea Fishery Commission (IBSF). Only from the early 1970s onwards Cod fishing started to be formally regulated in the Baltic Sea in line with developments on the international level coming from the UN conference on the sea in 1972. The establishment of EEZ made explicit and provided a strong institutional redefinition of marine resources as drivers for development. Seckinelgin (2009, p. 15) notes “by definition, the ecological identity, which is based on ecological life, of the resources is *subsumed under the raison d’état in relation to “development*””. The exploitation of marine resources thus became a driver for national development.

As a result of this raison d’état some Baltic States, for example Sweden, redefined an overall goal for fisheries from maintaining income levels approximately in line with agriculture (Period 1947–1969) to exploiting “water and fish resources in such a way that it can contribute to food supply and general welfare” (Period 1970–1994) which includes serving foreign markets (see Hentati-Sundberg et al., 2019). These periods are characterized by significant increases in government subsidies, mainly used for vessel construction and fishery development projects (Hentati-Sundberg et al., 2019). This contributed to the development and modernization of fishing fleets with increases in productivity, but where in the hunt for cod the individual fishing vessels were *de facto* transformed into “killing-machines” with dangerous overcapacities (Johnsen et al., 2009).

This was exacerbated by the international steering system put in place to manage the Baltic cod. The International Baltic Sea Fishery Commission (IBSF) recommended TAC figures for each year and the Baltic States negotiated corresponding national quotas. This system allowed “free access and competitive fishing until the total quota is taken, and then to close the fishery” (Holm and Nielsen, 2007). The hunt for cod was on and rewarded the most efficient and powerful cod capturing vessels. This was also known as “Olympic Fishing”, or “Race for Fish” (Hentati-Sundberg, personal communication) and this system created dangerous overcapacities, a dangerous excess of cod catching and killing capacity which induced well known non-compliance and cheating behavior Holm and Nielsen (2007). The Baltic cod stocks finally collapsed in the 1980s. Table 1 below summarizes the main elements of the discussion so far.

**TABLE 1:** Intra-active concepts and their definitions applied to the collapse of the Baltic cod in the 80s.

Intra-active concepts and their definitions…	…Applied to the collapse of the Baltic cod in the 80s
Phenomenon–The ontological inseparability of apparatus, objects, and agencies of observation	International Fisheries Management
Apparatus–Material-discursive arrangement that produce phenomena	The bifurcation of nature, models and approaches from natural resource economics, international relations and fisheries sciences understood as entangled, material-discursive practices which perform modern fisheries
Cut–Within a phenomenon a cut is realized where entities and their properties become determinate	Distinction between mind and matter, or subject and object. Nature is part of matter, reduced to its physicality and of value only in relation to subjects. This is disclosed by the whole array of technical/economic vocabulary and instruments that define modern fisheries
Territorializing processes stabilizing the cut and contributing to its further unfolding	Controllability, predictability (as part of the bifurcation of nature); increasing productivity (as part of the raison d´état of economic development)

## Discussion

### Intra-Active Intuitions

The core question of this paper concerns the causal contribution of the cut to the collapse of the cod in the Baltic sea. After having sketched out the material-discursive arrangements producing the cut, and the particular territorializing process–a drive for increasing productivity to realize economic development–that finally led to its collapse, how are we to answer this question? Rouse (2002) notes: “That a particular intra-action is causal indicates that under the right circumstances it’s pattern would recur”*.* Causality is thus expressed in terms of the repeatability of a material-discursive arrangement producing a phenomenon. The realization of the cut, however, is a singular event and therefore it is not only difficult to say whether the same material-discursive arrangement would have produced the same cut, but also to establish the role of the cut–as disclosed in the phenomena – in bringing about the collapse of the cod.

What we can say with some confidence is that the pursuit of development has had a negative impact on cod fisheries worldwide. The cod stocks of the North-West Atlantic, off the coast of Newfoundland, for example, collapsed within 15 years of the introduction of the Canadian Exclusive Economic Zone (EZZ). And looking beyond cod Hentati-Sundberg et al. (2019) generalize “many fisheries share the typical properties of overexploited natural resource systems”. The Food and Agriculture Organization of the United Nations (FAO, 2020) asserts that the percentage of stocks fished at biologically unsustainable levels is steadily increasing. Kituyi and Thomson (2018) highlight especially the role of fisheries subsidies, of which significant parts are channeled to increase productivity as part of the raison d’état of development: “There is no doubt that fisheries subsidies play a big role. Without them, we could slow the overexploitation of fish stocks, deal with the overcapacity of fishing fleets, and tackle the scourge of illegal, unreported and unregulated fishing” (Kituyi and Thomson, 2018). There might thus be some grounds to the claim that the processes of increasing productivity led to the collapse (not only of cod but to an overexploitation of fish stocks worldwide). And, given that we have argued that this process is *only intelligible* within this particular cut we might thus formulate the causal role of the cut in the collapse of the cod in the following way: The material-discursive apparatuses that produced the phenomenon of modern fisheries realized a particular cut. This cut provided a space of intelligibility within which a process of ever increasing productivity–as part of realizing the raison d’état of development–could unfold. This process is a major factor explaining the collapse of the Baltic cod. It is thus here that we abandon the familiar distinction between conditions and causes. What we usually conceive of as a cause can only meaningfully be understood to be a cause within a particular cut, thus drawing attention to the material-discursive arrangement that produces this very cut. This causal explanation is based on an intuition: Notably that such material-discursive arrangements, were they to realize elsewhere, would produce similar phenomena. It might seem unusual to associate terms like “intuition” and “causality” in this way, but causal explanations come in different shades and degrees.

The concept of intuition is controversial in philosophy and has received many definitions spanning from equating it to beliefs to describing it as some sort of emergent phenomenon beyond consciousness. Here we understand intuition in the bergsonian-deleuzian tradition. As Keith Robinson defines, intuition for Bergson is “a “reversal” of the normal workings of the intellect” (Robinson, 2009, p. 227). Intuition in this sense is an invitation to embrace the understanding of the world in process-relational terms, i.e., to try and grasp the flow of events, instead of separating components of phenomena for analysis.

This has consequences for what we normally mean when talking about a causal explanation. Separating components is an operation of the intellect and involves making abstractions with respect to particular properties of interest (e.g., related to the physicality of components). Proceeding in this way might make it easier to study these components and to come up with a causal explanation on the basis of a correct (i.e., validated according to some scientific standard) understanding of relations of dependency between those components. From the dominant scientific perspective, an explanation based on a sense, or feeling of understanding, nurtured for example by intuition, appears as limited (Ylikoski, 2009). Nevertheless it is a causal explanation, but it is crucial that one keeps in mind and recognizes it’s speculative nature. Albeit speculative, we argue that intuition has an important role to play in providing guidance for identifying causation. Swedberg (2012) argues that it is by the appeal to intuition that the “creative” dimension of an explanation can unfold. This might include an appeal to what is often labeled “unscientific”: “one needs inspiration, and to get inspiration one can proceed in whatever way that leads to something interesting” which could include as diverse things as feelings, observation, analogies, metaphors, etc. In this way intuition can create a space for *transcending* cut-internal explanations because it has the ability to escape the material-discursive framings that often orient scientific practice.

What is more, we argue that giving room to intuition might open up novel avenues not only for *explaining* but also for *transforming* a system. These transformations are not transformations designed within a cut (i.e., on the basis of a world of interactions) but transformations that aim at modifying the cut itself (i.e., on the basis of a world of intra-actions). Put differently, in a world where causality is understood in terms of repeatability instead of regularity we are presented with very different leverage points for transformation that furthermore normally receive less attention.

### Transforming the Cut

The literature on sustainability transformations is rich and diverse. Such transformations are said to encompass many phases and scales (Olsson et al., 2004; Moore et al., 2014). By identifying causal relationships–such as overfishing or changes in the cod environment–researchers open ways that suggest changes to lead the system to sustainable states. Posing that task specifically from a process-relational perspective would aim at realizing a different cut, at opening ways of determination that are different from existing ones. Leaving aside the current scientific paradigm to embrace the one sketched out above, which we can qualify as a process-relational one, is a notoriously difficult task (Hertz et al., 2020; Mancilla Garcia et al., 2020; West et al., 2020). In this section, we will try to translate some of the concepts proposed above to concrete actions that might constitute an entry point to such transformation.

We have discussed the hegemonic dominance of the material-discursive apparatus of modernity. Yet, as we saw with the example of the ayllu, there are pockets of resistance and contestation that offer different material-discursive apparatuses. Indeed, a key aspect put forward by process relational perspectives involves paying attention to local knowledge (Mason, 2002). Local knowledge has the characteristic of being tuned to context and therefore more resilient to the dangers of abstraction. Local knowledge might rely on unspoken or tacit aspects that can be revealed by confronting local and expert knowledge. Crucially, local knowledge might offer pathways for novel intuitions, i.e., ways to reverse established understandings of phenomena. Concretely, the recurrent organization of participatory processes building on methods such as, for example, the Multiple Evidence base (Tengö et al., 2014) have the potential to support inclusive governance that is aware of relations. This perspective can be integrated in new approaches to governance that would recurrently hold participatory meetings not only as decision-making fora but also as a source of qualitative indicators of change in the system that might, in turn, produce and stabilize different cuts. Holding these meetings in the Baltic might have revealed differences in observations at diverse localities that could have been followed up closer by managers.

Moreover, putting into dialogue different knowledge systems might allow revealing areas of unease or contested concepts (Mancilla García and Bodin, 2019). Unease in turn might pinpoint the presence of managerial aspects that are at odds with local understandings of the system. Finally, participatory processes can be complemented with work on scenarios in which participants might imagine pathways for intra-species co-existence.

In a word, participatory spaces open ways for co-existence that do not necessarily build on consensus but that navigate through different observations and perspectives. This has the potential of being highly informative on changes in the system. Indeed, the understanding of governance presented here resonates with the Deleuzian deterritoralization/reterritorialization couple. Deterritorializing supposes to dismantle the relationships that sustain existing arrangements and reterritorializing supposes establishing new ones. Understanding destabilizing and stabilizing processes as part of governance involves paying attention to which relationships are becoming loose or which are on the contrary, being strengthened. For example, the cod boom signified a change in relationships since it led to an increase in fishing. Observing the change in the intensity of the relationship “fishing” could have led to a search for explanations that would have contributed to deterritorializing the drive for increased productivity. In turn, it could have allowed the production of futures based on that change. It could have invited questions such as “what might this intensified type of fishing lead to? What might it mean for the sprat? For small-scale fishers? For the system?”.

Methods inspired in the arts have the potential to help imagine alternative pathways by providing a playful environment to explore the unexpected connections and reversed perspectives characteristic of bergsonian intuition. By engaging with methods such as collages, free word association or other art-based games, participants can see aspects that they did not see before. Complementing this with work recently developed among transformation scholars (O’Brien et al., 2019; Leichenko and O’Brien, 2019) and scholars in the environmental humanities (Haraway, 2016) such as future narratives, science-fiction narratives or theatre can help imagine diverse organizations of social-ecological systems and provide ground for inspiring change. Arts-based methods can also help develop empathy and an ability to see from the perspective of others (Morin, 2007). Others are not necessarily human others but can include species or specific individuals within species as well as networks. What would it have been like to imagine the future from the perspective of the cod or the sprat in the Baltic in the 1970s, before the collapse? What kind of material-discursives apparatuses would that have brought to the fore? Bringing together different types of knowledge might help identify interesting candidates to reflect about other positions.

As argued in this section, process-relational perspectives embrace a transdisciplinary perspective on knowledge generation and as such also propose a different engagement with governance. A process-relational governance does not offer a linear take on the links between knowledge and governance but much on the contrary understands them as intuitive threads of the same weaving process. For that reason, the boundaries that separate researcher and participants are very much blurred, which in turn changes the position of the researcher as the objective observer of exterior phenomena that are then transformed into knowledge. Instead, the researcher has an active role in shaping phenomena and the formerly observed have an active and explicit role in shaping a certain cut. This expands the capacities of the researcher by putting her in relation with others and therefore including relationalities such as those brought by emotions (Kleinman and Copp, 1993) and using them as a tool to navigate conversations and making explicit diverse positionalities.

## Conclusion

The previous section offered some ideas about ways and means that could help transcending existing cuts. This alone, however, might not be sufficient for transforming a cut. For a new cut to develop, to take hold and unfold it needs processes that stabilize it in its unfolding, i.e., its territorialization in Deleuzo-Guattarian terms - How can we think about such processes? What could they look like? A cut, as Debaise (2020) reminds us, is *political* because it reduces Being to particular experiences, and the processes of territorialization keep this understanding of Being in place. It is political because this begs the question: what interests *does* a particular cut serve? And whose interests *should* it serve? In her talk “Cosmopolitics: Learning to Think with Sciences, Peoples and Natures” Isabelle Stengers (Stengers, 2014) proposes to answer this question in terms of “relevance”. By relevance she means that the question of relevance should not be left to a selected few, but to embed and rethink relevance from within the concerns of the public (Stengers and Muecke, 2018). This might allow other experiences of Being to enter into play, realized by alternative material-discursive arrangements to the dominant ones, and taking us beyond experiences of Being in terms of its physicality. Relevance, thus understood, might serve as that which keeps the possibilities for understanding Being open. As an institutionalized criteria for scientific practice, it might provide the necessary space for other processes of territorialization to emerge. Novel cuts might emerge. Relevance, as a multi-faceted criteria, might then orient the unfolding of a cut - a process, as we have seen in the last section, which is in constant renegotiation. The understanding of causality put forward in this paper sheds light on a dimension that is often disregarded and that touches upon the core of the question of Being: In the end–and it might be good to be reminded about this–a discussion on causality has political implications: There are ways and means of not enclosing Being in a particular material-discursive arrangement. We should strive at keeping the possibilities for understanding Being open.

## Data Availability

The original contributions presented in the study are included in the article, further inquiries can be directed to the corresponding author.
